# Determinants of downloads and citations for articles published in intensive care medicine

**DOI:** 10.1186/2197-425X-3-S1-A863

**Published:** 2015-10-01

**Authors:** J-F Timsit, G Citerio, M Lavilloniere, A Perner, M Smith, S Ruckly, M Bassetti, J Bakker, D Benoit, JR Curtis, G Doig, M Herridge, S Jaber, L Papazian, M Peters, P Singer, M Soares, A Torres, A Viellard-Baron, E Azoulay

**Affiliations:** Medical and Infectious Diseases ICU, Universite Paris Diderot, Paris, France; Università degli Studi di Milano, Milano, Italy; Universite Paris Diderot, Paris, France; University of Copenhagen, Copenhagen, Denmark; The National Hospital for Neurology and Neurosurgery, University College London Hospitals, London, United Kingdom; Paris Diderot Sorbonne University, Paris, France; Ospedaliera Universitaria Santa Maria della Misericordia, Udine, Italy; Erasmus MC University Medical Center, Rotterdam, Netherlands; Ghent University Hospital, Ghent, Belgium; The University of Washington, Seattle, United States; University of Sydney, Sydney, Australia; University of Toronto, Toronto, Canada; Saint Eloi University Hospital, Montpellier, France; Aix-Marseille Universite, Marseille, France; Institute of Child Health and Great Ormond St Hospital, London, United Kingdom; Tel Aviv University, Tel Aviv, Israel; D'Or Institute for Research and Education, Rio de Janeiro, Brazil; Hospital Clınic of Barcelona, Barcelona, Spain; Hospital Ambroise Paré, Boulogne, France

## Introduction

Medical publications undergo a comprehensive process of editor's handling, peer reviewing and editing that intends to select manuscripts that are both likely to be read and cited.

## Objectives

This study is part of a global quality improvement process for articles published in Intensive Care Medicine.

## Methods

All papers accepted in 2012-2013 have been tracked through the Web-of-Science database for referencing and the Springer link statistics report for downloads from 01/01/2013 to 12/31/2014. Relative risk of being downloaded or cited one time are modeled with a multiple negative binomial regression.

Variables tested were submitting country, manuscript category, open access, key-words, topics, number of author, and H-index of first and last author.

## Results

Among the 404 articles, 304(61%) were original (including 59 pediatric), 46(11.5%) were review articles (including 10 Conference Reports and Expert Panel papers), and 32(8%) were experimental. Major topics were sepsis (21%), ventilation(20%) and hemodynamic(16%). Only 6% of the papers were in open access. The median (IQR) number of authors per articles was 7 (5-9), with H-index of first and last authors of 9(4-16) and 23(15-37), respectively. The total number of 2013-2014 downloads was 696[467-1083] and the total number of 2013-2014 cites was 6 (4-11) per article.

Independent predictors of downloads included five groups of variables. Namely, the second trimester of each year (RR = 1.31(1.06-1.63)) for 2012 and 1.29(1.07-1.57) for 2013), manuscript's keyword with “septic shock” (RR 1.57(1.22-2.02)); manuscript type (Conference Reports and Expert Panel, RR 20.4 = (13.79-30.2); original (vs. experimental), RR = 1.97 (1.61-2.41), review articles, RR 4.28 (3.31-5.52), and what's new papers 3.18 (2.35-4.31). Open access papers were significantly more downloaded (RR 1.49 (1.18-1.87)). Last, the H index of the last author was significantly associated with the number of downloads (H-index>37, RR 1.2(1.04-1.40)).

Independent predictors of cites included: manuscript type (Conference Reports and Expert Panel, RR 4.6(2.52-8.41), review articles, 3.55(2.39-5.28), and original manuscripts, RR 1.8(1.23-2.63); Number of authors (7-9 (RR = 1.24(1.01-1.52)), >9, RR 1.5(1.21-1.87)); and H index of the first author (4-9, RR = 1.21 (1.01-1.45), 10-16, RR = 1.35 (1.1-1.65), and >16, RR = 1.36(1.12-1.66). Open access papers were more likely to be cited (RR 1.31 (1.01-1.70)).

## Conclusions

This study provides key elements to improve our understanding of what makes a paper read or cited. Optimizing use and identification of manuscript's keywords appears as a simple and major way to improve access to ICM articles. Seasonal download variation by overcommitted critical care specialists underline the need to increase the access of our articles by the use of press release, tweets and take home messages through social and specialized media. Last, possibility for articles of being open accessed improved their impact.

